# Hospitalization and surgery rates in patients with inflammatory bowel disease in Brazil: a time-trend analysis

**DOI:** 10.1186/s12876-021-01781-x

**Published:** 2021-04-27

**Authors:** Flávia Gonçalves Musauer Palacio, Lucila Marieta Perrotta de Souza, Jéssica Pronestino de Lima Moreira, Ronir Raggio Luiz, Heitor Siffert Pereira de Souza, Cyrla Zaltman

**Affiliations:** 1 Departamento de Clínica Médica, Hospital Universitário, Universidade Federal Do Rio de Janeiro, Rua Prof. Rodolpho Paulo Rocco 255, Ilha Do Fundão, Rio de Janeiro, RJ 21941-913 Brazil; 2 Instituto de Estudos de Saúde Coletiva (IESC), Universidade Federal Do Rio de Janeiro, Rio de Janeiro, 21944-970 Brazil; 3 D’Or Institute for Research and Education (IDOR), Rua Diniz Cordeiro 30, Botafogo, Rio de Janeiro, RJ 22281-100 Brazil

**Keywords:** Inflammatory bowel disease, Crohn`s disease, Ulcerative colitis, Hospitalization rates, Surgical rates, Lethality

## Abstract

**Background:**

The prevalence of inflammatory bowel disease (IBD) is increasing globally, and the disease is frequently managed surgically. The aim of this study was to investigate the time trends and geographic distribution of IBD hospitalizations, surgeries and surgical-associated lethality.

**Methods:**

Data from the Brazilian Health Public System were retrospectively collected regarding hospitalizations, in-hospital deaths, IBD-related surgical procedures and lethality from 2005 to 2015.

**Results:**

This eleven-year period revealed decreases in the rates of hospitalization (24%), IBD-related surgeries (35%), and IBD-related surgical lethality (46%). Most surgeries were performed in Crohn’s disease patients, and the predominant procedure was small bowel resection, mostly in young adults. A higher prevalence of ulcerative was observed throughout the country. The highest hospitalization and surgical rates were observed in the more industrialized regions of the South and the Southeast and in the municipalities integrated with metropolitan regions (MRs). The highest surgical-related lethality rates were seen in the less-developed regions and in municipalities not integrated with MRs. The length of hospital stay showed a slight increase throughout the period.

**Conclusions:**

Brazil follows the global trend of decreases in hospitalizations, lethality, surgeries, and surgical lethality associated with IBD. The unequal distribution of hospitalizations and surgeries, concentrated in the industrialized areas, but with a shift towards the Northeast and from urbanized to rural areas, indicates ongoing changes within the country. Reductions in the rates of IBD-related hospitalizations, surgeries and lethality suggest the effectiveness of decentralization and improvements in the quality of public health services and the advances in medical therapy during the study period.

**Supplementary Information:**

The online version contains supplementary material available at 10.1186/s12876-021-01781-x.

## Background

Crohn’s disease (CD) and ulcerative colitis (UC), the two major forms of inflammatory bowel disease (IBD), constitute chronic inflammatory conditions of the gastrointestinal tract with a multifactorial etiology, characterized by a relapsing and remitting course [1–3]. Initially described in more developed and industrialized countries (mostly in Europe and North America) [4, 5], their incidence has been increasing worldwide in the last few decades [6], including in newly industrialized countries, such as Brazil and other South American countries [7–10].

Disease activity, drug availability and clinical response to medical therapy are known to influence the outcomes of these conditions [11, 12], but the appropriate management of accumulated structural damage may require surgical interventions [13–16]. It has been estimated that 10–30% of patients with UC [1, 17] and approximately 38–70% of patients with CD will undergo surgery in the first 10 and 20 years after the diagnosis has been established, respectively [1, 17]. For CD, the estimated chance of surgery throughout the patient’s lifetime has been reported to be 70–80% [13, 18, 19]. This might considerably affect the patient’s quality of life and might also impose a relevant socioeconomic impact on patients and a burden to healthcare systems [20, 21], especially considering that IBD frequently originates in young adults [22].

Despite the increasing incidence and prevalence rates of IBD, recent information supports the idea that the dramatic advances in treatment, especially in the last two decades, with new drugs and therapeutic strategies [23], have had a remarkable impact on the overall surgical interventions and hospitalization rates [24–27].

However, few data exist concerning surgery, hospitalization and lethality related to IBD. Therefore, this study aimed to analyze trends of hospitalizations and IBD-related surgical procedures in Brazil. In particular, we evaluated the geographic distribution and demographic aspects in an attempt to identify associations and risks related to the geographic regions and urbanization patterns.

## Methods

### Data source

Data available from the Health Informatics Department/Brazilian Ministry of Health (DATASUS) (http://www2.datasus.gov.br/DATASUS) were retrospectively retrieved, similar to previous studies from our group addressing gastrointestinal malignancies [28–30]. In the DATASUS homepage, health information is available through the tabulation software TABNET, with access to Epidemiology and Morbidity data (http://www2.datasus.gov.br/DATASUS/index.php?area=0203&id=6926).

DATASUS is an open access population-based health and disease registry that contains information from the Unified Health System (SUS, Sistema Único de Saúde) on medical procedures, hospital admission and discharge, mortality, and demographic variables and encloses roughly the whole population. All data are anonymous and do not allow identifying individual subjects. Currently, Brazil’s SUS is one of the largest public health systems in the world, and SUS hospital beds account for almost 75% of the total number of hospital beds (Additional file 1: Fig. S1). Records of hospitalizations for IBD obtained from the DATASUS registry were searched according to the International Statistical Classification of Diseases and Related Health Problems, Tenth Revision (ICD-10). The ICD-10 codes considered for the search were as follows: K50.0 to K50.9 for CD and K51.0 to K51.9 for UC. To select patients who were submitted to surgery related to IBD, we used codes of procedures described in the hospitalization authorization forms (Additional file 1: Tables S1 and S2). Those codes are numerical sequences standardized by the Brazilian Ministry of Health, and they correspond to specific hospital-based procedures.

### Study design, population, and variables

An ecological study was performed retrospectively with DATASUS records for hospitalizations due to IBD from January 2005 through December 2015, with data analysis and time-series graphs, allowing us to project temporal trends. The period of study was selected based on the most recent and standardized data available. The period of time analyzed in this study was chosen by convenience. The systematic insertion of standardized data in the database begun 2005, and by the time this study was carried out, data were available only to the end of 2015.

Information presented on hospitalization forms includes age, sex, municipality of origin of the hospital unit, procedures performed during hospitalization and type of outcome (discharge or death). Age groups were stratified by every 20 years: 0–19 years, 20–39 years, 40–59 years and 60 years or more. Variables analyzed were age, sex, municipality of origin of the hospital unit, procedures performed during hospitalization, length of stay, and type of outcome (discharge or death).

To define geographic distribution, we utilized two criteria: division by macroregions (North, Northeast, Southeast, South, and Central-west) and division by municipalities, according to Veiga’s model [31]. For the analysis of the geographic distribution, IBD hospitalization rates were calculated per 100,000 inhabitants in each municipality. Surgical rates and associated lethality were calculated utilizing the total hospitalizations due to IBD per year as the base population.

### Statistical analysis

Exploratory procedures with a quantitative approach were applied to the data using IBM SPSS software for Windows (Version 20, SPSS Inc., Chicago, IL, United States), allowing a temporal trend analysis. Descriptive summary statistics and graphical displays were generated by Tabwin 3.2 (Tab for Windows 3.2, free software that allows organization of multiple applications into grouped tabs, available at http://www2.datasus.gov.br/DATASUS). IBD hospitalization rates were adjusted by the general available hospital beds in Brazil during the study period (Additional file 1: Fig. S1). Estimates of resident populations were obtained from the Instituto Brasileiro de Geografia e Estatística (IBGE; Brazilian Institute of Geography and Statistics) [32]. Simple linear regression was used to estimate temporal trends in IBD-related surgeries and the associated lethality by age and sex. Categorical variables were described as absolute frequencies (n) and proportions (%), whereas continuous variables were characterized by their medians and percentiles, namely, interquartile range (IQR). Graphs were generated using Microsoft Excel Software (Microsoft Excel for Mac 2011, Version 14.4.9, 2010; Microsoft Corp, Redmond, Wash).

## Results

### Hospitalizations for IBD

**Fig. 1:**
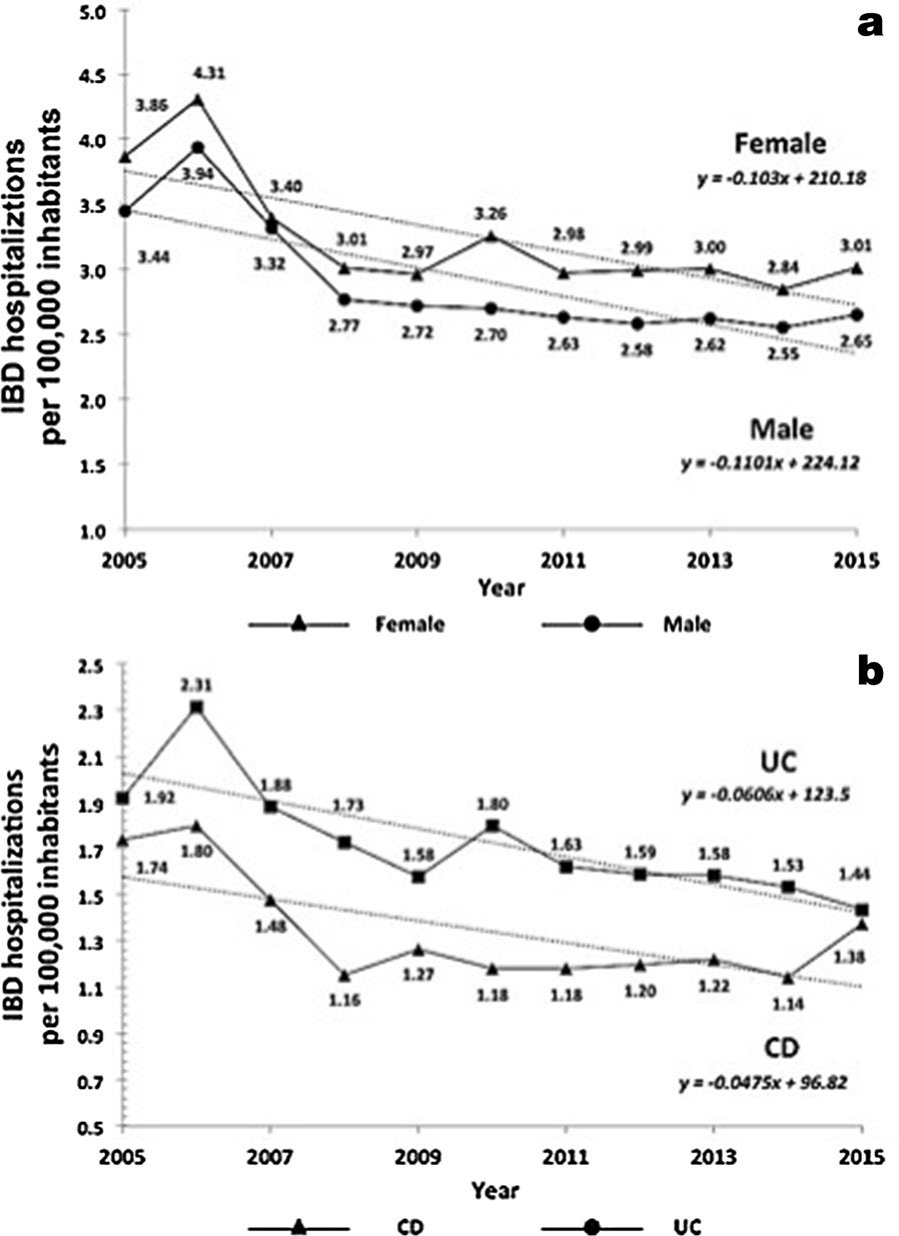
Hospitalizations for inflammatory bowel disease (IBD) per 10,000 inhabitants in Brazil from 2005 to 2015, and distributions according to sex (**a**) and diagnosis of ulcerative colitis (UC) or Crohn’s disease (CD) (**b**)

From 2005 to 2015, 47,699 hospitalizations due to IBD were registered in DATASUS, including 26,883 for UC and 20,816 for CD. IBD hospitalizations adjusted to the totality of available hospital beds in Brazil decreased 24% in the period, from 3.72 per 100,000 inhabitants in 2005 to 2.83 per 100,000 inhabitants in 2015. Regarding sex, hospitalization rates for females were higher than those for males (ratio 1.13:1), and they decreased essentially in parallel (Fig. 1a). Hospitalization rates per 100,000 inhabitants were invariably higher for patients with UC than for patients with CD, at 1.92 and 1.74 in 2015 and 1.44 and 1.38 in 2015, respectively, and decreased in parallel (Fig. 1b).

**Fig. 2:**
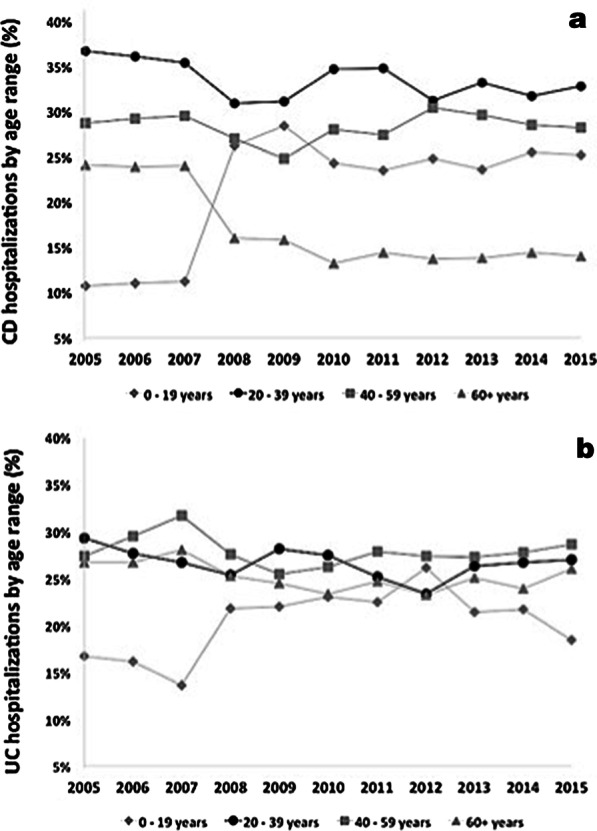
Hospitalizations for Crohn’s disease (CD) (**a**) or ulcerative colitis (UC) (**b**) in Brazil from 2005 to 2015, by age ranges

Further analysis revealed that hospitalizations for CD were proportionally higher among patients aged 20–39 years, whereas in patients with UC, the highest proportion of hospitalizations was among patients aged 40 years or older. Although hospitalizations remained stable across most age ranges, we observed an increase among patients aged 0–19 years in IBD, more so in patients with CD (Fig. 2).

During the study period, the average length of hospital stay for patients with IBD was 7.1 days (SD 9.2, IQR 3–8), being 7.3 days (SD 9.8, IQR 2–8) for CD and 6.9 days (SD 8.7, IQR 3–8) for UC, respectively. Both groups of patients registered increases in the average length of hospital stay from 2005 to 2015. For CD, the increase was from 6.9 to 7.6 days, whereas for UC, it was from 6.9 to 7.2 days. As expected, the length of stay was higher for surgical-related hospitalizations (11 days, SD 11.3; IQR 5–13), and similar values were registered for CD and UC. The length of stay for surgical patients with UC increased from 10.9 in 2005 to 15.2 in 2015 (Additional file 1: Table S3).

### IBD-related surgical procedures, associated mortality and their trends

**Fig. 3:**
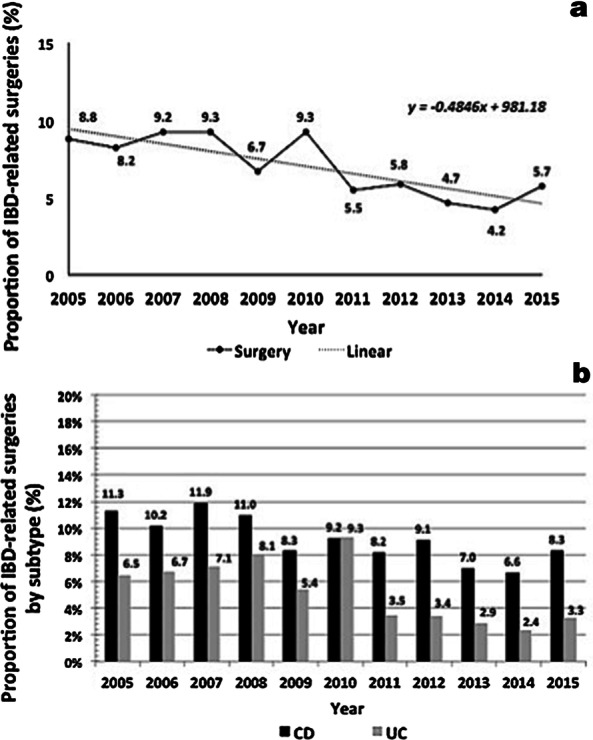
Overall proportions of inflammatory bowel disease (IBD)-related surgeries (**a**) and according to the diagnosis of Crohn’s disease (CD) or ulcerative colitis (UC) (**b**) in Brazil from 2005 to 2015

The overall rate of IBD-related surgeries decreased 35.2% during the study period, from 8.8% (445 surgeries/5070 hospitalizations) in 2005 to 5.7% (235 surgeries/4109 hospitalizations) in 2015 (Fig. 3a). Operative rates were higher in CD patients during the whole period, from 2005 (61.3%) to 2015 (70.6%). Small bowel resection was the most often performed surgery (45.8% of the total) (Additional file 1: Table S4), although it showed a reduction from 56.6% of the total of surgeries in 2005 to 41.7% in 2015. The highest surgical rates were registered in CD hospitalizations throughout the period analyzed. Temporal analysis revealed a decreasing number of surgical procedures for both IBD types (Fig. 3b).

**Fig. 4:**
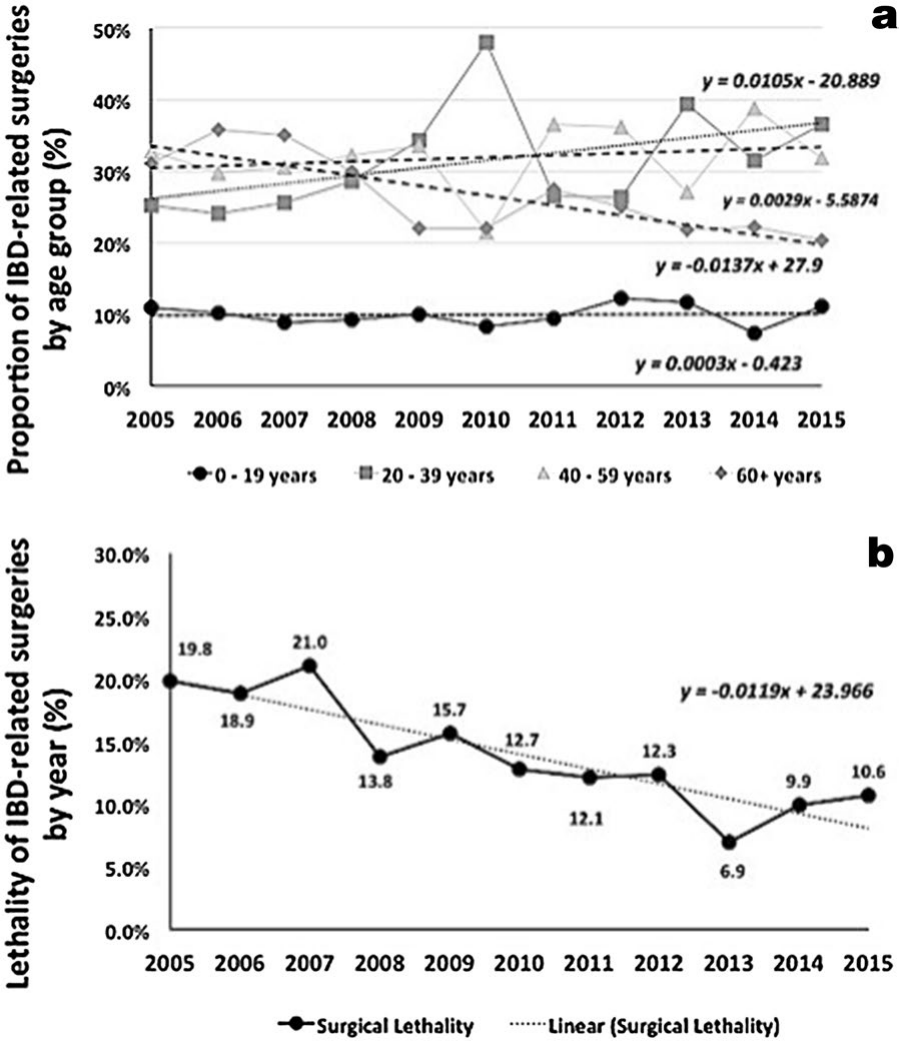
Overall proportions of inflammatory bowel disease (IBD)-related surgeries by age ranges (**a**) and lethality rates of inflammatory bowel disease (IBD)-related surgeries (**b**) in Brazil from 2005 to 2015

Altogether, patients aged 40–59 years had the highest proportion of surgical procedures in the period (approximately 33%). However, throughout 2005 to 2015, there was an increase of 48% in the total number of surgeries performed in patients aged 20–39 years, a trend not observed in other age groups (Fig. 4a). Compared to females, males presented a slightly higher proportion of surgeries in the period (53.7% in 2005 and 50.6% in 2015) (Additional file 1: Fig. S2).

In-hospital lethality of IBD surgical patients decreased from 19.8% in 2005 to 10.6% in 2015 (− 46%) (Fig. 4b). During the same period of time, the general in-hospital lethality rate regarding SUS beds increased from 3.1 to 4.1 (Additional file 1: Fig. S3).

### Geographic distribution of IBD hospitalizations and surgeries

The Southeast and South regions, the most developed regions in the country, registered the highest adjusted hospitalization rates for IBD. Hospitalization rates for IBD decreased 24% from 2005 to 2015, mainly in the North and Northeast regions, which presented the highest rates of reduction (− 43% and − 44%, respectively). Conversely, an increase of 10% in IBD hospitalization rates was observed in the South. Considering in-hospital lethality of IBD patients for all regions, our study indicates a decrease of 13%, although the North and Northeast regions displayed a trend towards increasing lethality rates (Table 1).

**Table 1:** Hospital admission and lethality rates for patients with IBD, by macro-region

	2005			2015				
Geographic regions	Population	Adjusted admission rate (per 10^5^)	Lethality (%)	Population	Adjusted admission rate (per 10^5^)	Lethality (%)	Changes in adjusted admission rates (%)	Changes in lethality (%)
Brazil	184,184,074	3.72	3.1	204,450,380	2.83	2.7	− 24	− 13
North	14,698,834	3.63	0.5	17,472,646	2.09	3.9	− 43	676
Northeast	51,018,983	3.12	2.8	56,560,034	1.74	3.4	− 44	24
Southeast	78,472,036	3.92	3.8	85,745,427	3.20	2.4	− 18	− 36
South	26,973,432	3.96	2.6	29,230,070	4.34	2.1	10	− 19
Central-west	13,020,789	3.60	4.0	15,442,203	2.74	3.7	− 24	− 8

The overall proportion of surgical procedures in IBD hospitalizations decreased 35% during the study period (from 8.8% in 2005 to 5.7% in 2015). Most surgical procedures took place in the Southeast and South regions, where the registered rates were above the national average (CD: 12.2% in 2005 and 6.5% in 2015; UC: 9.2% in 2005 and 3.9% in 2015). Over the 11-year period analyzed, a decrease of 46% in lethality could be detected among IBD patients who underwent surgical procedures, notably in the Central-west and Southeast regions (− 77 and − 62%, respectively), while the North region showed an increase of 60% (Table 2).

**Table 2:** Proportion of surgeries and surgical lethality of IBD patients, by macro-region

	2005			2015			Changes in proportion of surgeries (%)	Changes in surgery lethality (%)
Geographic regions	Proportion of surgeries (%)	IBD hospitalized population	Surgery lethality (%)	Proportion of surgeries (%)	IBD hospitalized population	Surgery lethality (%)		
Brazil	8.8	5070	19.8	5.7	4109	10.6	− 35.2	− 46
North	2.0	402	12.5	1.9	259	20.0	− 3.0	60
Northeast	4.8	1198	22.8	7.0	700	14.3	47.1	− 37
Southeast	12.2	2313	18.4	6.5	1949	7.1	− 46.6	− 62
South	9.2	804	14.9	3.9	901	17.1	− 57.8	15
Central-west	6.8	353	45.8	6.3	300	10.5	− 6.8	− 77

Taking into account Brazilian disparities, hospitalizations, lethality and surgeries data were stratified according to municipality size and relationship to MR. Regarding this relationship, adjusted hospitalization rates in 2005 were higher in municipalities not integrated with MR (4.3 vs 3.0 per 100,000); the rates were quite similar in 2015 (2.9 vs 2.8 per 100,000). Municipalities not integrated with MR had a 33% average decrease in IBD hospitalization rates and a 21% decrease in lethality, mainly driven by large-sized municipalities (a 47% decrease in hospitalization and a 39% decrease in lethality in the period) (Table 3). During the period, the rates of surgical procedures decreased, mainly in large-sized municipalities not integrated with MRs, except for small-integrated municipalities, which registered a 177% increase. Surgical-related lethality showed a consistent reduction throughout the country, except in small-integrated municipalities (+ 33%), which represent approximately 5% of the population living in municipalities integrated with MRs (Table 4).

**Table 3:** Hospital admission rates and lethality in IBD patients, by municipalities

Municipalities	2005			2015			Changes in adjusted admission rates (%)	Changes in lethality (%)
	Population	Adjusted admission rate (per 10^5^)	Lethality (%)	Population	Adjusted admission rate (per 10^5^)	Lethality (%)		
*Integrated to MR*	91,248,203	3.0	2.7	98,192,651	2.8	2.7	− 8	0
Small-sized	4,542,857	4.0	2.9	3,919,865	3.6	4.0	− 12	38
Medium-sized	9,013,588	2.7	1.1	9,429,076	2.8	2.7	2	145
Large-sized	77,691,758	3.0	2.8	84,843,710	2.7	2.6	− 8	− 7
*Not integrated to MR*	92,934,991	4.3	3.4	106,257,729	2.9	2.7	− 33	− 21
Small-sized	46.661.002	3.6	2.4	49,253,779	2.9	2.6	− 20	8
Medium-sized	22.965.072	4.8	2.8	26,762,001	3.0	2.5	− 37	− 11
Large-sized	23.308.917	5.4	5.1	30,241,949	2.8	3.1	− 47	− 39

MR, metropolitan region

**Table 4:** Proportion of surgeries and surgical lethality of IBD patients, by municipalities

Municipalities	2005		2015		Changes in proportion of surgeries (%)	Changes in surgery lethality (%)
	Proportion of surgeries (%)	Surgery lethality (%)	Proportion of surgeries (%)	Surgery lethality (%)		
*Integrated to MR*						
Small-sized	2.19	0.00	6.06	33.33	177	33
Medium-sized	7.57	14.28	5.91	9.09	− 22	− 36
Large-sized	9.16	17.09	6.06	9.09	− 34	− 47
*Not integrated to MR*						
Small-sized	7.02	23.59	3.45	11.42	− 51	− 52
Medium-sized	7.32	20.00	5.30	13.33	− 28	− 33
Large-sized	12.93	21.48	4.26	19.23	− 67	− 10

MR, metropolitan region

## Discussion

In this study, we describe, for the first time to our knowledge, information on hospitalizations and surgical treatment for IBD in Brazil over a recent period of time. Data obtained from an official source of the Ministry of Health indicated that the overall IBD hospitalizations decreased twenty-four percent, while surgical treatment decreased thirty-five percent, and the lethality of IBD-related surgeries decreased almost fifty percent during this eleven-year period. The geographic distribution of IBD hospitalizations showed an overall higher prevalence of UC, with greater concentrations of hospitalizations in the South and the Southeast, where surgery rates decreased more, particularly in municipalities not integrated with MRs. Nevertheless, during the same period, lethality rates associated with IBD hospitalizations and IBD-related surgeries increased in the North region, more so in small- and medium-sized municipalities integrated with MRs.

Hospitalization rates per 100,000 inhabitants, adjusted to the totality of available hospital beds, were invariably higher for patients with UC compared to CD, and they decreased essentially in parallel during the period. Hospitalization rates for females were slightly higher. Some Brazilian regional studies indicate a slight predominance of females among the patients with IBD, but with no significant differences [10, 33, 34], similar to the world literature [4]. Further analysis revealed that hospitalizations for CD were proportionally higher among patients aged 20–39 years, whereas in patients with UC, the highest proportion of hospitalizations was among patients aged 40 years or older. Although hospitalizations remained stable across most age ranges, we observed an increase among young patients, more so in CD. These results appear to be in accordance with most international data showing that usually UC peaks first, followed by CD, with a lapse of approximately a decade [5, 35]. In addition, CD preferentially affects younger individuals, whereas UC occurs in relatively older patients, with a characteristic bimodal distribution, with second peak after age 40 [36].

Temporal analysis revealed generally decreasing numbers of surgical procedures for both IBD types. Nevertheless, CD presented a higher proportion of surgeries than UC, which is also in agreement with the literature, since several studies have shown that CD has a cumulative risk of surgery higher than UC [37–39]. Altogether, patients aged 40–59 years had the highest proportion of surgical procedures in the period. However, from 2005 to 2015, there was an increase of almost fifty percent in the total number of surgeries performed in patients aged 20–39 years, a trend not observed in other age groups. This may have occurred due to the probable increase in the number of new cases emerging in younger individuals and the likely increase in the number of more complex and severe forms of disease as IBD progresses in the country. In fact, the increase in the number of surgeries among younger patients observed in this study also might be related to the global increase in IBD prevalence corroborated by recent studies carried out in the country (7, 33, 51).

Regarding length of hospital stay, both groups of patients registered an increase in the average length of hospital stay from 2005 to 2015. For CD, the increase was from 6.9 to 7.6 days, whereas for UC, it was from 6.9 to 7.2 days. As expected, the length of hospital stay was higher for surgical-related hospitalizations, with similar values for CD and UC. While the findings of this study are agreement with data from some European countries [40, 41], they are in contrast with others [24], probably indicating the heterogeneity of the populations affected and their respective health systems. Nonetheless, in the period analyzed here, the in-hospital lethality of IBD surgical patients decreased to almost half, whereas the general in-hospital lethality rate regarding SUS beds increased approximately thirty percent. These findings suggest that although the reduction in the number of available beds imposes a selection of the most severe cases, and probably longer hospital stays, the overall outcomes regarding IBD-related hospitalizations and surgical procedures apparently improved.

It is important to highlight the fact that even with the reduction of the in-hospital lethality of IBD surgical patients to 10.6% observed in this study, the numbers are higher than the ones from other studies, ranging from 2.5 to 6.0% [42–44]. Such discrepancies may reflect differences in the efficiency of the respective health systems where data originate from, including availability of medication, access to specialists, and the time lapse before diagnosis. However, it is likely that such disparities also may reflect differences in study designs, which renders data difficult to compare. For example, some investigations analyze data from elective surgeries, while others include emergency procedures [43, 45, 46]. On the other hand, some studies focus on the status of medical therapy and the use of biological agents, while others on the presence of comorbidities in the study population, or the duration of the disease and specific surgical procedures [42–47]. Moreover, some studies may consider both intra- and extra-hospital post-operatory mortality, from one month up to three years after surgery [42, 46, 47]. The databank used in the present study does not allow the analysis of all these individual variables, what may contribute to the apparently higher rates detected.

The most frequently identified profile among the surgical patients was CD (71% in 2015), with a slight predominance of males and a predominant age range of 20–59 years (40–59 years in 2005, and 20–39 years in 2015). The predominance of young male patients (not including the pediatric population, which has the lowest surgery rates) has also been described before [37]. The most common type of surgical procedure was major surgery, with abdominal laparotomy, which may be related to the longer length of hospital stay for surgical patients. Surgeries involving the small bowel, especially resections and treatment of complications of CD (e.g., fistulas) were the most commonly performed procedures, in agreement with the higher rates of surgeries observed in CD patients (small bowel involvement).

In assessing the country according to its macro regions, the highest hospitalization rates and the largest proportions of surgeries were concentrated in the South and Southeast, which could be justified by the fact that these are the most developed regions of the country, with more equipped hospitals, that are associated with large MRs. During the study period, the in-hospital lethality of IBD patients decreased, whereas the lethality of surgical patients had an even more marked reduction. Together, these findings may reflect improvements in the care of patients with IBD, including pre-and postoperative management, and the technical and quality care in the centers where most of these procedures took place.

Since Brazil is a country with continental proportions and great heterogeneity regarding demographic and socioeconomic characteristics, in this study, we also analyzed data from the smallest administrative unit, the municipalities. The analysis of municipalities showed an overall reduction in hospitalization rates, but the reduction was greater for those not integrated with MRs. This finding may reflect recent improvements in health care in the latter, tending to technically approach the care offered in large centers in 2015. Following this line of reasoning, we observed a reduction in lethality of hospitalized patients in municipalities not integrated with MRs, while practically no changes were observed in the integrated municipalities. However, when assessing surgical treatment, we note that large municipalities associated or not with MRs present the highest proportions of surgeries, and large ones associated with MRs maintained the highest rate of all in 2015. A possible explanation is the concentration of IBD referral centers in these areas. In contrast to the overall reduction in IBD-related surgeries, small municipalities associated with MRs showed an increase, suggesting a tendency towards the higher concentration of these procedures in more developed regions, such as those associated with MRs. This may also indicate a possible migratory movement of patients from underresourced areas to referral hospitals. However, there was also an increase in proportions of surgeries in small municipalities, where large, specialized centers are not normally allocated. This may reflect a trend towards decentralization of surgeries, although still in greater concentration in more developed regions of the country. Corroborating this fact, we observed that the lethality of operated patients was higher in municipalities not associated with MR, especially in small ones, where there are probably fewer resources and qualified professionals for more complex procedures.

The last two decades witnessed the progressive development of new drugs and diagnostic tools for IBD, an increased early referral to specialists, and changes related to diagnostic and treatment strategies, such as combined drug therapy with immunosuppressants and biologics [48, 49]. In this scenario, accumulated information has led to the interpretation of a probable relationship between these changes in patient management and the improvement in major outcomes, notably with fewer surgeries [50, 51] and, in some populations, fewer hospitalizations [39, 52]. However, there is a lack of studies with appropriate follow-up data to confirm these direct relations, particularly in the long term [25, 53]. Furthermore, it has been suggested that the decrease in surgical rates occurs mostly due to emergency procedures, with no significant changes in elective procedures [54]. The results from this study are in agreement with international trends, showing progressive reductions in surgeries and hospitalizations, including surgical hospitalizations, and decreased surgery-related lethality among the patients with IBD throughout the country [23, 55, 56]. The widespread use of immunosuppressants, followed by the introduction of biologic therapies in the public health system in the last decade, might have had an important impact on the overall outcomes of patients with IBD in the country, as pointed out previously [57]. Nevertheless, temporal association with our findings may not constitute a sufficient explanation. Even before biologic therapy availability, hospitalizations and surgeries were already reducing, which led us to think of other possible factors, such as improvements in diagnosis and patient follow-up. In a previous study from our group, using a databank from the social security benefits, we demonstrated that IBD frequently leads disability for prolonged periods and contributes to early retirement, but with clear reduction trends between 2010 and 2014 in the country [20]. Although the databank used in the current study does not contain information on the socioeconomic or work status of the patients, the general trend towards reductions in hospitalizations and IBD-related surgeries follow a similar pattern and appear to be in accordance with reductions in work disability, previously observed. Taken together, these data appear to support the notion of probable ongoing improvements in the management of IBD in the country.

Although the findings presented here offer insights into time trends and the geographic distribution of IBD hospitalizations and IBD-related surgeries and lethality for the first time in Brazil, several limitations of this study need to be addressed. The inherent characteristics of this type of study and the possibility of an ecological fallacy [58] have been greatly diminished due to the application of an objective and consistent methodology, allowing a straightforward analysis of the electronic data entered in a single national database. However, the large amount of data from the whole country in this database does not have specific information on disease details, medical follow-up or comorbidities. Moreover, hospital-based documentation does not cover outpatient procedures and medical therapy. Another potential caveat of this study involves the concept of the municipality. Typically diverse and heterogeneous, Brazil has cities with broad differences in terms of population densities. For example, some municipalities may display populations ranging from one thousand to twenty million inhabitants and would still be classified on the same level in the database. However, an analysis based on municipalities may compensate for system defects, such as underreporting or equivocal registry, because the single and official database covers the whole country in its minimal administrative units.

## Conclusions

The analysis of hospitalizations, surgical treatment, and the associated lethality due to IBD in Brazil has revealed a general trend towards reductions in hospitalizations and IBD-related surgeries, particularly in the more developed regions and large municipalities integrated with MRs. This trend was less pronounced in the less-developed regions, where the overall IBD-related lethality and IBD-related surgical lethality increased. Regardless of macroregions, municipalities not integrated to MRs, harboring almost half of the population, also showed decreases in both hospitalizations and lethality related to IBD, suggesting probable improvements in health care beyond the limits of the MRs. In this regard, particularly with the advent of biological therapy in Brazil after 2007, advances in the medical treatment might have influenced the reductions in hospitalizations and IBD-related surgeries. Nevertheless, these data also suggest that a national program for optimizing the care of patients with IBD should reinforce the process of decentralization, as critical for the favorable outcomes of patients, probably reducing the need for hospitalization and surgical treatment. However, the higher lethalities associated with IBD-related surgeries in the less-developed regions and in small- and medium-sized municipalities, even when integrated with MRs, appear to indicate the need for improving the care of complex and severe cases in these locations. In the context of an increasing prevalence of IBD, professional multidisciplinary training and further development of excellence centers that could provide access to a similar level of care for every patient should be pursued by the authorities to guide improvements in the existing system, as should changes to the current health policies.

## Supplementary Information

**Additional file 1.** Supplementary materials.

## Data Availability

Data from the Health Informatics Department of the Brazilian Ministry of Health (DATASUS) are freely available on the Internet at http://www2.datasus.gov.br/DATASUS. DATASUS registries include hospital admission and discharge information, medical procedures and mortality, reference tables and demographic data (age, sex, municipality) collected by the Instituto Brasileiro de Geografia e Estatística (IBGE; Brazilian Institute of Geography and Statistics).

## Abbreviations

IBD: Inflammatory bowel disease
CD: Crohn’s disease
UC: Ulcerative colitis
ICD-10: International Classification of Diseases 10th Revision
MR: Metropolitan region
