# Supplementary material for: Hospitalization and surgery rates in patients with inflammatory bowel disease in Brazil: a time-trend analysis
Source: BMC Gastroenterol. 2021 Apr 27;21:192. doi: 10.1186/s12876-021-01781-x (PMC8077865; doi:10.1186/s12876-021-01781-x)
Supplement: Supplementary file 1 — Additional file 1. Supplementary materials. [file 12876_2021_1781_MOESM1_ESM.docx]

**Additional file 1**

Additional file 1: Table S1. Surgical procedures codes list until 2007

| Year | SUS code | Procedure |
| --- | --- | --- |
| Until 2007 | 33006067 | partial colectomy (hemicolectomy); |
|  | 33007063 | total colectomy; |
|  | 33050066 | vídeo laparoscopic colectomy; |
|  | 33003068 | proctocolectomy |
|  | 33022062 | abdominal rectosigmoidectomy; |
|  | 33026068 | abdominoperineal rectosigmoidectomy |
|  | 33032114 | pelvic abscess drainage; |
|  | 33008078 | incision and drainage of perianal abscess; |
|  | 33040060 | excision and drainage of submucosal abscess of rectum |
|  | 33021066 | small bowel resection |
|  | 33046069 | laparoscopic small bowel resection |
|  | 33008060 | enteroanastomosis (any segment) |
|  | 33009066 | enteropexy (any segment) |
|  | 33012067 | enterotomy (any segmente) |
|  | 33010064 | enterorrhaphy (any segment) |
|  | 33045062 | laparoscopic enterorrhaphy |
|  | 33002061 | abdominoperineal resection of the rectum |
|  | 33023069 | colostomy; |
|  | 33723060 | colostomy |
|  | 33047065 | laparoscopic colostomy |
|  | 33027064 | ileostomy |
|  | 33049068 | laparoscopic ileostomy |
|  | 33007071 | fistulectomy / anal fistulotomy |
|  | 31021018 | fistulectomy |
|  | 33005079 | anal fistulectomy |
|  | 34011021 | surgical treatment of rectovaginal fistula |
|  | 31016030 | surgical treatment of enterovesical fistula |
|  | 31021034 | surgical treatment of rectovesical fistula |
|  | 33018065 | colon fistula closure |
|  | 33019061 | rectal fistula closure |

Additional file 1: Table S2. Surgical procedures codes list from 2008 onwards.

| Year | SUS code | Procedure |
| --- | --- | --- |
| From 2008 | 407020063 | partial colectomy (hemicolectomy) |
|  | 407020071 | total colectomy |
|  | 407020080 | vídeo laparoscopic colectomy; |
|  | 407020330 | total proctocolectomy with ileoanal reservoir |
|  | 407020403 | abdominal rectosigmoidectomy; |
|  | 407020411 | abdominoperineal rectosigmoidectomy |
|  | 407040013 | pelvic abscess drainage |
|  | 407020179 | small bowel resection |
|  | 407020187 | enteroanastomosis (any segment) |
|  | 407020195 | enteropexy (any segment) |
|  | 407020209 | enterotomy and/or enterorrhaphy with suture / resection (any segment) |
|  | 407020012 | abdominoperineal resection of the rectum |
|  | 407020101 | colostomy |
|  | 407020306 | ileostomy / jejunostomy |
|  | 407020136 | anorectal abscess drainage |
|  | 407020276 | fistulectomy / anal fistulotomy |
|  | 409070238 | surgical treatment of rectovaginal fistula |
|  | 409010456 | surgical treatment of enterovesical fistula |
|  | 409010464 | surgical treatment of rectovesical fistula |
|  | 407020250 | colon fistula closure |
|  | 407020268 | rectal fistula closure |

Additional file 1: Table S3. Length of stay for IBD hospitalizations and related surgeries

|  |  | **2005-2015** | **2005** | **2015** |
| --- | --- | --- | --- | --- |
| **CD** | Hospitalizations |  |  |  |
|  | (N) | 20,816 | 2,409 | 1,997 |
|  | LOS (days), median (IQR) | 7.3 (2-8) | 6.9 (2-7) | 7.6 (2-9) |
|  | Surgeries |  |  |  |
|  | (N) | 1,946 | 273 | 166 |
|  | LOS (days), median (IQR) | 11.0 (5-13) | 11.2 (5-14) | 11.2 (4-13) |
| **UC** | Hospitalizations |  |  |  |
|  | (N) | 26,883 | 2,661 | 2,112 |
|  | LOS (days), median (IQR) | 6.9 (3-8) | 6.9 (3-8) | 7.2 (3-8) |
|  | Surgeries |  |  |  |
|  | (N) | 1,475 | 172 | 69 |
|  | LOS (days), median (IQR) | 11.1 (5-13) | 10.9 (5-14) | 15.1 (5-20) |

CD, Crohn’s disease; UC, ulcerative colitis; LOS, length of hospital stay; IQR, inter-quartile range.

Additional file 1: Table S4. Type of surgical procedures in absolut number, 2005-2015.

| TYPE OF SURGERY | N |
| --- | --- |
| small bowel resection | 1567 |
| total colectomy | 1302 |
| enterotomy and/or enterorrhaphy | 253 |
| proctocolectomy | 158 |
| colon fistula closure | 87 |
| fistulectomy / anal fistulotomy | 22 |
| partial colectomy (hemicolectomy) | 17 |
| abdominal rectosigmoidectomy | 5 |
| colostomy | 3 |
| ileostomy / jejunostomy | 2 |
| rectal fistula closure | 1 |
| enteroanastomosis | 1 |
| abdominoperineal rectosigmoidectomy | 1 |
| surgical treatment of rectovaginal fistula | 1 |
| abdominoperineal resection of the rectum | 1 |
| TOTAL | 3421 |

Additional file 1: Figure S1. Number and distribution of hospital beds in Brazil from 2005 to 2015, considering SUS and non-SUS beds.


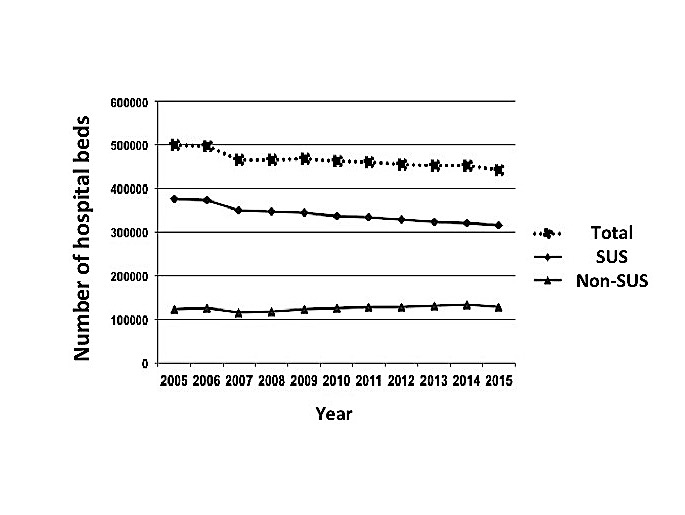


Additional file 1: Figure S2. Proportion of inflammatory bowel disease (IBD)-related surgeries in Brazil from 2005 to 2015, among males (A) and females (B).

~~
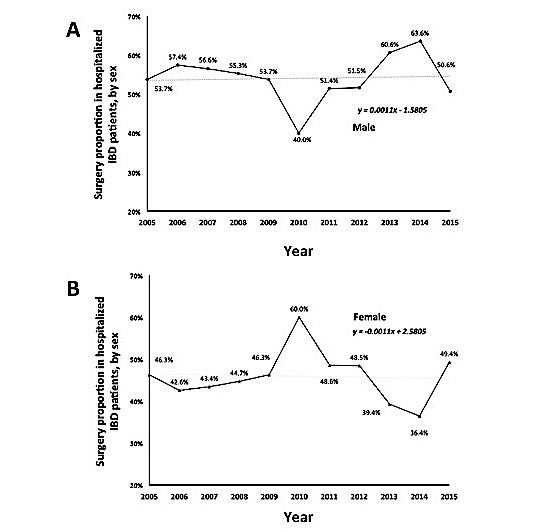
~~

Additional file 1: Figure S3. General lethality rate in SUS beds in Brazil from 2005 to 2015.

~~
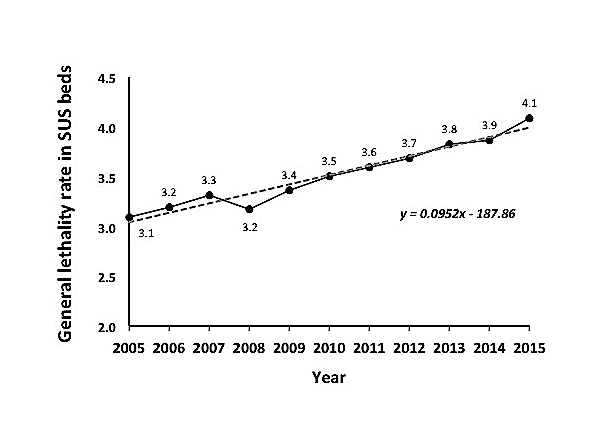
~~
